# Evaluation of NO_x_ emissions of a retrofitted Euro 5 passenger car for the Horizon prize “Engine retrofit”

**DOI:** 10.1016/j.envres.2018.06.006

**Published:** 2018-10

**Authors:** Barouch Giechaskiel, Ricardo Suarez-Bertoa, Tero Lähde, Michael Clairotte, Massimo Carriero, Pierre Bonnel, Maurizio Maggiore

**Affiliations:** a European Commission – Joint Research Centre, via E. Fermi 2749, I-21027 Ispra, VA, Italy; b European Commission – DG Research & Innovation, ORBN 07/139, B-1049 Brussels, Belgium

**Keywords:** Air pollution, Vehicle emissions, Nitrogen oxides, Real driving emissions, Solid ammonia, Selective catalytic reduction (SCR)

## Abstract

The Horizon 2020 prize for the “Engine Retrofit for Clean Air” aims at reducing the pollution in cities by spurring the development of retrofit technology for diesel engines. A Euro 5 passenger car was retrofitted with an under-floor SCR (Selective Catalytic Reduction) for NO_x_ catalyst in combination with a solid ammonia based dosing system as the NO_x_ reductant. The vehicle was tested both on the road and on the chassis dynamometer under various test cycles and ambient temperatures. The NO_x_ emissions were reduced by 350–1100 mg/km (60–85%) in the laboratory depending on the test cycle and engine conditions (cold or hot start), except at type approval conditions. The reduction for cold start urban cycles was < 75 mg/km (< 15%). The on road and laboratory tests were inline. In some high speed conditions significant increase of ammonia (NH_3_) and nitrous oxide (N_2_O) were measured. No effect was seen on other pollutants (hydrocarbons, carbon monoxide and particles). The results of the present study show that retrofitting high emitting vehicles can significantly reduce vehicle NO_x_ emissions and ultimately pollution in cities.

## Highlights

• Many studies report diesel vehicles emitting more than 4 times the type approval NO_x_ in real driving conditions.
• One solution is retrofitting them, but there is no legislative background for passenger cars.
• The Horizon 2020 prize for the “Engine Retrofit for Clean Air” aims at reducing the pollution in cities.
• A Euro 5 retrofitted vehicle showed significant reductions over test cycles, but small at the cold start phases.
• In some high speed conditions NH_3_ and N_2_O were measured.

## Graphical abstract

**Figure:**
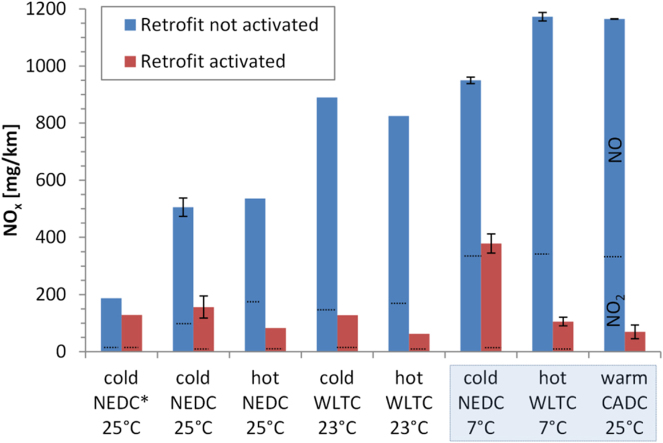

## Acronyms

ADAC: General German Automobile Club
APC: AVL (Austria) Particle Counter
Artemis: Assessment and Reliability of Transport Emission Models and Inventory Systems
ASC: Ammonia Slip Coating
ASDS: Ammonia Storage and Delivery System
CADC: Common Artemis Driving Cycle
CAN: Controller Area Network
CLD: Chemi-Luminiscence Detector
CO: Carbon monoxide
CO_2_: Carbon dioxide
DEF: Diesel Exhaust Fluid
DOC: Diesel Oxidation Catalyst
DPF: Diesel Particulate Filter
EGR: Exhaust Gas Recirculation system
EH: Extra High
EU: European Union
EUDC: Extra Urban Driving Cycle
FID: Flame Ionization Detector
FTIR: Fourier-Transform Infrared Spectroscopy
ISC: In-Service Conformity
JRC: Joint Research Centre
N_2_O: Nitrous oxide
NDIR: Non-Dispersive Infrared Detectors
NEDC: New European Driving Cycle
NH_3_: Ammonia
NO: Nitrogen oxide
NO2: Nitrogen dioxide
OBD: On-Board Diagnostics
PEMS: Portable Emissions Measurement System
PM: Particulate Matter
PN: Particle Number
RDE: Real-Driving Emissions
REC: Retrofit Emission Control
SCR: Selective Catalytic Reduction
THC: Total Hydrocarbons
TUG: Technical University of Graz
UDC: Urban Driving Cycle
UNECE: United Nations Economic Commission for Europe
VERT: Verification of Emission Reduction Technologies
WLTC: World Harmonised Light-duty Transient Cycle
WLTP: World Harmonised Light-duty Test Procedure

## 1 Introduction

Air pollution is recognized as an important global health risk factor. In Europe the most serious pollutants in terms of harm to human health are particulate matter (PM), nitrogen dioxide (NO_2_) and ground-level ozone (O_3_) (EEA, 2017). NO_2_ is associated with both short-term and long-term adverse health effect (WHO, 2013, Faustini et al., 2014). In 2015 around 10% of all the reporting stations in Europe recorded concentrations above the NO_2_ limit values; almost 90% of all concentrations above this limit value were observed at traffic stations (EEA, 2017). The transport sector is the largest contributor to NO_2_ emissions, accounting for 39% of total NO_2_ emissions in the European Union (EU) in 2015 (EEA, 2017). The estimated impacts of exposure to NO_2_ concentrations in Europe in 2014 were around 78.000 premature deaths (EEA, 2017).

Diesel vehicles have high emissions of nitrogen monoxide (NO) and NO_2_, collectively known as nitrogen oxides (NO_x_). Most of the NO_x_ in vehicle exhaust was associated to NO; however, recent technologies produce a high percentage of NO_2_ (Weiss et al., 2011) in order to promote low temperature oxidation of soot (Ehrburger et al., 2002) at the Diesel Particulate Filters (DPF) and higher efficiency of Selective Catalytic Reduction (SCR) systems (Goo et al., 2007). NO is oxidized to NO_2_ in the atmosphere, which is one reason why they are reported together as NO_x_. The NO_x_ emissions from vehicles in Europe are regulated through Euro standards, which were first introduced in 1990s. The vehicles are tested in the laboratory following a prescribed procedure. The Euro 5 limit, introduced in 2009, is 180 mg/km and the Euro 6, introduced in 2014, is 80 mg/km for diesel light-duty vehicles. However, it was found that in some cases the Real-Driving Emissions (RDE) are much higher than the type approval values (Weiss et al., 2011). Based on 541 diesel cars, Euro 5 vehicles were on average 4.1 times higher than the Euro 5 limit and Euro 6 vehicles were 4.5 times the Euro 6 limit (Baldino et al., 2017). The main reasons for such discrepancy are: a) the type approval procedure, which was not always reflecting real world driving, b) the “thermal-windows” and the so called “defeat devices”, such as “cycle detection“, that reduce the effectiveness of the emission control system under some conditions (Degraeuwe and Weiss, 2017).

Sales of new passenger cars in the EU in 2017 were more than 15 million, which is at the same level as in the years before the economic crisis in 2007 (ICCT, 2017b). For the last 15 years the market share of diesel vehicles in EU is 45–55%. A study estimated that there are around 29 million high emitting (defined as > 3 times higher than the type approval limit) Euro 5 and Euro 6 diesel passenger cars and vans on the European roads; about 76% of all diesel vehicles registered over the 5 years assessed (2011–2015) (Transport and Environment, 2016). These cars are expected to be on the road for 10 – 15 years contributing to the air pollution. The impact of excess NO_x_ emissions could be at least halved if diesel vehicles respected their type approval limit also on the road (Jonson et al., 2017). Another study estimated that the traffic-influenced stations exceeding the air quality limit for annual mean NO_2_ could be reduced from about 50% in 2015 to 8% in 2025 and 1% in 2030 if the RDE emissions will respect the type approval with a conformity factor of 1.5 (Toenges-Schuller et al., 2016).

In May 2018 the European Commission referred France, Germany, and the United Kingdom to the EU Court of Justice for failure to respect limit values for nitrogen dioxide (NO_2_), and for failing to take appropriate measures to keep exceedance periods as short as possible (EC, 2018). Cities have already started implementing Low Emissions Zones (CLARS, 2018). Ambient measurements showed that long term average Particulate Matter (PM) and NO_2_ concentrations have been reduced by a few percent at such zones (Holman et al., 2015). Based on a landmark court ruling, German cities will be allowed to ban older diesel vehicles from some areas. Other cities, like Paris, Madrid, Mexico City and Athens might ban diesel vehicles from city centres by 2025 (BBC news, 2018). On 1 April 2018, the Vehicle Excise Duty (car tax) in the UK changed for diesel cars (VCA, 2018): any new diesel car that does not comply with Euro 6d is required to pay tax at the next higher band. However, diesel vehicles have lower CO_2_ emissions and fuel consumption. A solution to air pollution from high emitting diesel vehicles is retrofitting them. For passenger cars the experience is limited. Tests carried out by the regional branch of the General German Automobile Club (ADAC) in the state of Baden-Wurttemberg showed that the on-road emissions of Euro 5 diesel cars can be reduced by 70–90% if the vehicles’ hardware is adjusted appropriately (ADAC, 2018). The experience for heavy-duty applications is much more profound (see Kubsch (2017) for a review). The Verification of Emission Reduction Technologies (VERT) industry association introduced retrofit protocol for NO_x_ reduction devices a few years ago (Czerwinski et al., 2014). Since 2014, Regulation 132 from United Nations Economic Commission for Europe (UNECE) covers the approval of Retrofit Emission Control (REC) devices for heavy duty vehicles, agricultural and forestry tractors and non-road mobile machinery equipped with compression ignition engines (UNECE, 2014, EU, 2018).

The Horizon prize for the “Engine Retrofit for Clean Air” (2016–2018) (EC, 2016) aims at reducing the pollution produced by the existing passenger cars fleet by spurring the development of retrofit-able technology (i.e. additional devices and/or modifications) applicable to diesel engines. The focus was on NO_x_ emissions of Euro 5 light-duty vehicles under real driving conditions, but other pollutants were considered, such as particles, N_2_O and ammonia (NH_3_) due to their effect on human health (WHO, 2013), climate change (IPCC et al., 2014) and ecological effect (Behera et al., 2013), and particle formation in the atmosphere (Updyke et al., 2012), respectively. Additionally, vehicle fuel efficiency, retrofitting costs, durability, maintenance, usability, safety, drivability, and noise were taken into account in the prize criteria. The implementation of the technology should allow such retrofitted vehicles to circulate without unduly affecting air quality. It is expected that a successful application of clean engine retrofits will improve health and life quality of European citizens.

## 2 Horizon 2020: Engine retrofit for clean air

This section summarizes the rules of the Horizon 2020 “Engine retrofit for clean air” prize of 1.5 million Euros (EC, 2016).

The retrofit technology had to be installed on a mass production Euro 5b C-class compact car in the top C-class sales, but limited to high-volume hatchback and three volumes family car bodies. The engine had to be compression ignition using diesel (not natural gas or gasoline) without any hybridization. The permitted vehicles and engines were chosen to achieve the maximum impact for the overall emissions of the European fleet.

The vehicle should retain most of its payload carrying capability, but the retrofit was allowed to reduce boot volume by 20 litres. No modification to the body, the chassis and the auxiliaries of the donor vehicle was permitted. The standard gearbox of the donor vehicle had to be left unmodified. A limited amount of hybridization (peak power for 30 s up to 20% of the internal combustion engine’s power, maximum continuous power up to 10% of the internal combustion engine power and up to 0.5 kW h of additional energy storage) was allowed. The use of additives or on-board generated chemicals was permitted. In all cases the retrofit should not affect the safety or the homologation potential.

For the submission of an application some pollutants thresholds had to be met by the retrofitted vehicle; the pre-submission tests had to be conducted in an external laboratory. The most demanding thresholds were NO_x_ emissions below 180 mg/km, less than 10% fuel consumption increase, and maximum retrofit and consumables costs for 100,000 km of 2000 Euros.

The prize was to be awarded to the application that, in the opinion of the jury and according to testing at the European Commission Joint Research Centre (JRC), demonstrated a solution that best addressed the following criteria:

● Levels of NO_x_ (< 180 mg/km) and NO_2_ emissions (< 60 mg/km and < 35% of NO_x_).
● Levels of solid particle number (PN) (< 6 × 10^11^ particles/km) and PM emissions (< 4.5 mg/km).
● Levels of emissions of other pollutants, including but not limited to total hydrocarbons (THC) (THC+NO_x_ < 230 mg/km), carbon monoxide (CO) (CO< 500 mg/km), nitrous oxide (N_2_O) (< 40 mg/km), ammonia (NH_3_) (< 60 mg/km).
● Fuel consumption (< 10% increase).
● Performance and driveability (accelerations less than 20% worse).
● Acquisition and running costs (< 2000 Euros).
● Noise and safety.
● Durability, maintenance and usability.

Each criterion gave points depending on the performance of the vehicle with and without the retrofit. The pollutants were scored based on the mean of three test cycles (see below Section 3) in the laboratory and checked with Real-Driving Emissions (RDE) tests on the road.

A retrofit technology could additionally be awarded extra points if it could decrease the NO_x_ emissions of Euro 6 vehicles to the applicable limit with a conformity factor of 2.1 (i.e. 168 mg/km) or if it could be applied to a car without particle filter bringing it to the Euro 6 PN limit.

The contest was launched on the 20th of April 2016 with a registration deadline of 12th June 2017. Participants had to submit the application and deliver the prototype/s (i.e. the donor vehicle with the installed innovation) to the JRC in Ispra by September 27th, 2017. The jury evaluation took place between October 2017 and March 2018. The chassis dynamometers and RDE tests took place in the same time period.

From the 9 registered submissions, only three delivered retrofitted vehicles. One of them had NO_x_ emissions higher than the threshold. The other one had low NO_x_ reduction (< 100 mg/km) at the JRC tests vs. better results in contestant's tests, for unknown reasons. The official testing of the third retrofitted technology installed on a Euro 5, along with additional testing will be presented in this paper. The same retrofit was installed additionally on a Euro 6 vehicle, but the JRC results didn’t confirm the reduction of NO_x_ emissions < 168 mg/km and thus the Euro 6 retrofitted vehicle was not awarded and will not be presented here. Nevertheless investigations are still going on. The prize award took place on April 16th, 2018 (Maggiore, 2018).

## 3 Materials and methods

### 3.1 Vehicle and retrofit device

The donor vehicle was a Euro 5b certified Renault Megane 1.5 dCi 110, year 2014 station wagon 1461 cm^3^, 81 kW, 72,000 km, 1383 kg (empty, without retrofit), with winter tires, 195/65 R15. The original exhaust configuration consisted of Exhaust Gas Recirculation system (EGR), Diesel Oxidation Catalyst (DOC) and Diesel Particulate Filter (DPF).

**Fig. 1:**
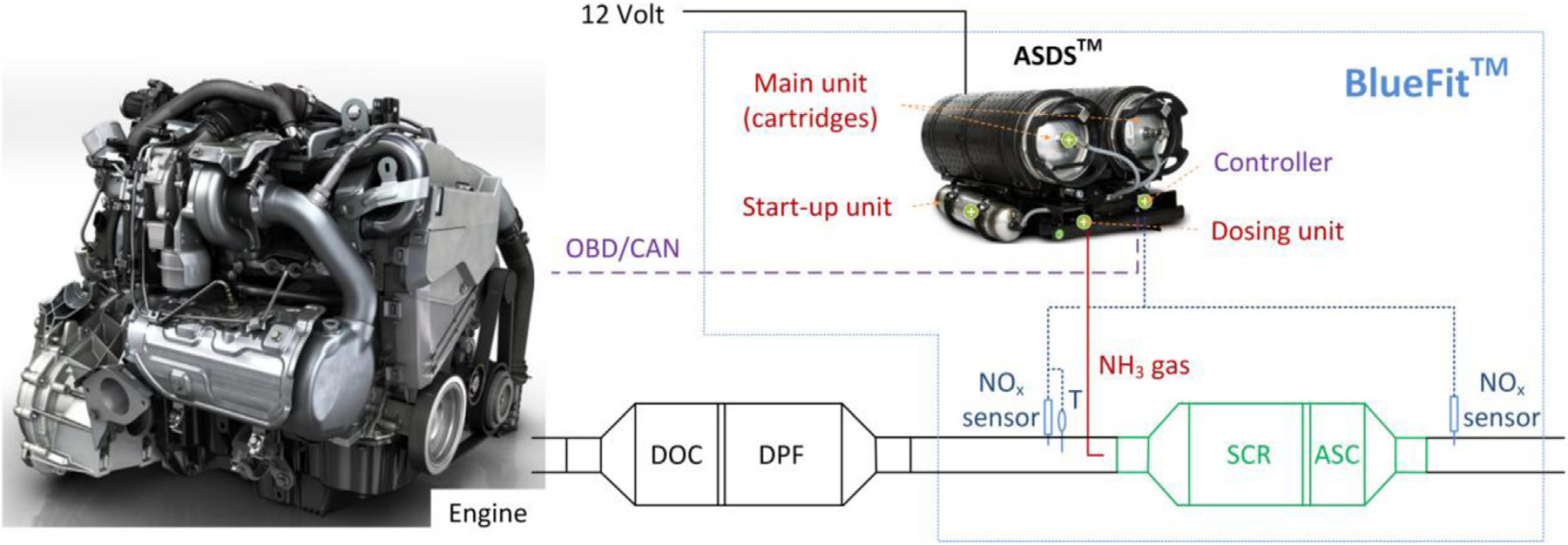
Retrofit (BlueFit™) configuration for the Euro 5 vehicle. The ASDS™ illustrated here is the variant for commercial vehicles. “T” for temperature sensor.

The retrofit, BlueFit™ (from a consortium including Amminex Emissions Technology A/S, of which Faurecia acquired 91.5% share, Johnson Matthey, Technical University of Graz (TUG) and the International Council on Clean Transportation Europe (ICCT)) comprises an Ammonia Storage and Delivery System (ASDS™) (Johannessen et al., 2008), mounted in the spare wheel well, and a commercially available underfloor Cu-Zeolite SCR catalyst with Pt-containing Ammonia Slip Coating (ASC), installed downstream of the DPF (Fig. 1). The technical approach of the consortium was to avoid any reconstruction of or interference with the type-approved hardware and adding the SCR function downstream of the original emissions components. Consequently, this configuration can be considered “stand-alone” and does not require interference with the engine, neither any exchange of already existing emissions hardware, as was required by the prize rules.

The ASDS™ prototype for passenger cars consisted of two 1.2 l AdAmmine™ cartridges, where ammonia is absorbed in strontium chloride salt in a solid form (Bialy et al., 2015) (the commercial unit will be 4.5 l), a start-up unit, which also contains AdAmmine, but in a much smaller volume (0.5 l) to enable fast dosing (for cold start), the dosing unit, which provides dynamic dosing of ammonia, and finally a controller with software. The prototype is a down-sized version of the system that is currently in production for commercial vehicle segment and in operation for emissions upgrade of bus fleets in e.g. Copenhagen and London.

In order to release ammonia, cartridges are equipped with electric heaters. During the engine cold start, most of the electrical power goes to the start-up unit to warm it up fast and enable ammonia dosing shortly after the engine start. The remaining power is then directed to one of the main cartridges. The system is ready to dose when the start-up unit has reached the target desorption pressure. The dosing strategy is based on NO_x_ measurements upstream and downstream of the SCR (with NO_x_ sensors), the exhaust mass flow, and the measured temperature upstream of the SCR. For the specific solution, the only output from the vehicle itself, which is important for the dosing strategy, was the exhaust mass flow rate from the Controller Area Network (CAN) bus, accessed through the On-Board Diagnostics (OBD) port.

The net system’s weight (without AdAmmine™ cartridges) was 10 kg. Each cartridge (main unit) weighted 3.1 kg, whereas the start-up unit 1 kg.

Compared to the water based solution of urea (Diesel Exhaust Fluid (DEF), AdBlue^®^ or AUS 32) the gaseous ammonia can be introduced into the exhaust gas already from a temperature of 100–140 °C in order to have high NO_x_ reduction efficiency. This temperature is lower than the minimum exhaust gas temperature of around 200 °C that is required for the injection of urea solution to ensure a complete decomposition and hydrolysis of urea to ammonia and to avoid catalyst deactivation, by-products and deposits (Brack et al., 2016, Guan et al., 2014, Lacin et al., 2011, Kim et al., 2014). While AdAmmine™ uses a formulation based on strontium chloride as carrier material, other solids have been considered in the past such as solid urea, ammonium salts (ammonium carbamate and ammonium carbonate), and other metal ammine chloride salts (magnesium ammine chloride, calcium ammine chloride, and strontium ammine chloride). Solid storage materials generally provide an equivalent number of moles of NH_3_ to urea solution in roughly half to one third the volume or the mass (Johannessen et al., 2008, Lacin et al., 2011).

### 3.2 Chassis dynamometer tests

The vehicle was tested at the Vehicle Emission Laboratory (VELA 2) of the European Commission Joint Research Centre (JRC), in Ispra, Italy. The climatic test cell temperature was kept at 23–25 °C or 7 °C with relative humidity of 50%.

**Fig. 2:**
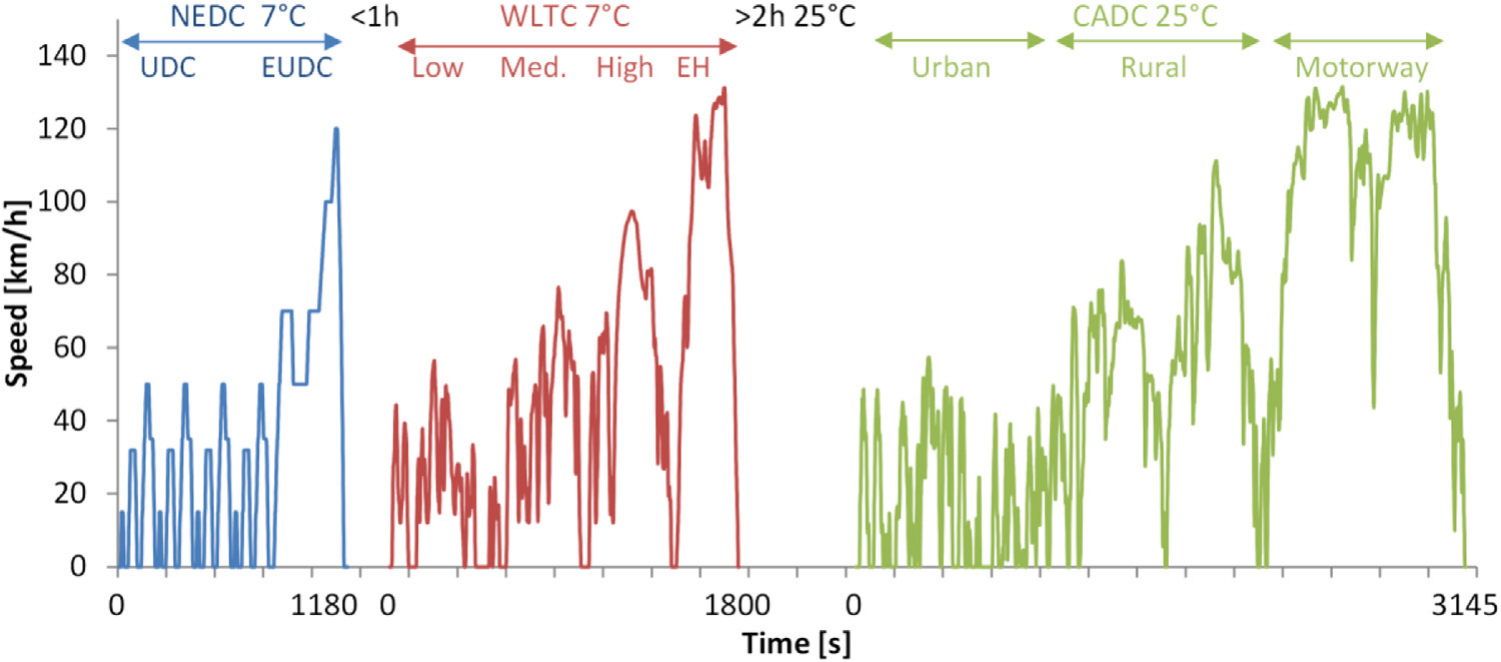
Test cycle and official test sequence. EH=Extra High.

The test protocol according to the prize rules was (Fig. 2):

● New European Driving Cycle (NEDC) with engine cold start at 7 °C climatic room temperature after overnight soaking at 7 °C and vehicle battery fully charged. The NEDC consists of the urban part (UDC) and the extra urban part (EUDC) (UNECE, 2015). The NEDC with cold start and ambient temperature at 20–30 °C was the type approval cycle in EU until September 2017.
● World Harmonised Light-duty Transient Cycle (WLTC) (Tutuianu et al., 2015) with engine hot start (i.e. oil temperature > 50 °C) at 7 °C climatic room temperature within one hour from the end of the NEDC test (no further charging of the battery). The WLTC consists of four phases (low, medium, high and extra high). In this paper the low phase is considered as the urban part. The WLTC with cold start at 23 °C is the new type approval cycle in EU since September 2017.
● Common Artemis Driving Cycle (CADC) at 25 °C after the climatic room has been stabilised for at least 2 h (no charging of the battery). The CADC was developed within the European Artemis (Assessment and Reliability of Transport Emission Models and Inventory Systems) project, based on statistical analysis of a large database of European real world driving patterns (André, 2004).

Additional NEDC and WLTC test cycles were conducted at 25 °C or 23 °C respectively with engine cold and/or hot start in order to check the legislated test cycles and gain better insight on the efficiency of the retrofit with cold and hot engine start. The three official tests were conducted at least twice with the retrofit deactivated and 3 times with the retrofit activated. The other tests were conducted once or twice. In order to avoid any influence of the precedent (or pre-conditioning) cycle on the results, only repetitions that were following identical procedures were compared.

The 2-axle dyno was set in rear wheels “following” speed mode. The chassis dynamometer parameters were selected according to the rules of the prize based on the UNECE Regulation 83 (UNECE, 2015) roller dynamometer coefficients using the empty weight of the donor vehicle minus any replaced or removed components plus 100 kg plus the retrofit weight (for the post retrofit tests). The dyno coefficients used were: m= 1470 kg, F_0d_= 7.4 N, F_2d_= 0.0502 N/(km/h)^2^. Only for one cold NEDC at 25 °C the estimated type approval values road coefficients were used (after dyno road load derivation): m= 1470 kg, F_0 s_ = 93.1 N, F_1 s_ = 0.371 N/(km/h), F_2 s_ = 0.0282 N/(km/h)^2^ (Tsiakmakis et al., 2017).

It should be mentioned that the retrofit was always installed on the car: its efficiency was evaluated by activating and deactivating it. Thus, the true vehicle emissions without the retrofit device installed could be different. Nevertheless, the presence of the SCR (without ammonia release) is expected not to have a negative effect compared to the original configuration. In addition, the data provided by the contestant done before installing the retrofit were in good agreement with JRC’s tests (Amminex, 2018).

Measurements of CO_2_, CO (both with Non-Dispersive Infrared Detectors, NDIR), NO_x_ (Chemi-Luminiscence Detector, CLD) and total HC (Flame Ionization Detector, FID) were taken from the tailpipe, and the diluted gas in the full dilution tunnel in real time (Nakamura and Adachi, 2013). Measurements from bags that were filled during the test, as described in the regulation, were also taken for every test. The gas analysers were the MEXA 7000 series from Horiba, Japan. The solid particle number system measuring solid particles > 23 nm connected at the full dilution tunnel was an AVL (Austria) Particle Counter APC 489 (Giechaskiel et al., 2010). A 10 nm Condensation Particle Counter (model 3772 from TSI, USA) was connected to measure additionally sub-23 nm particles.

Additional pollutants, including ammonia (NH_3_) and nitrous oxide (N_2_O) were measured with a Fourier-Transform Infrared Spectroscopy (FTIR) instrument (Sesam i60 from AVL) connected to the vehicle tailpipe, using a heated polytetrafluoroethylene sampling line (191 °C) (AVL, 2009). The FTIR instrument (Nicolet Antaris IGS Analyser - Thermo Electron Scientific Instruments LLC, Madison, WI, USA) was equipped with a multipath gas cell of 2 m of optical path, a downstream sampling pump (6.5 lpm flowrate) and had the acquisition frequency of 1 Hz with a working pressure of 860 hPa. The dispersive element of both FTIR instrument was made up of a Michelson interferometer (spectral resolution: 0.5 cm^-1^, spectral range: 600–3500 cm^-1^), while the detection was achieved with a liquid nitrogen cooled mercury cadmium telluride detector. The FTIR NO and NO_2_ data were used to calculate the NO_2_/NO_x_ (in ppm) ratios.

### 3.3 On-road tests

**Fig. 3:**
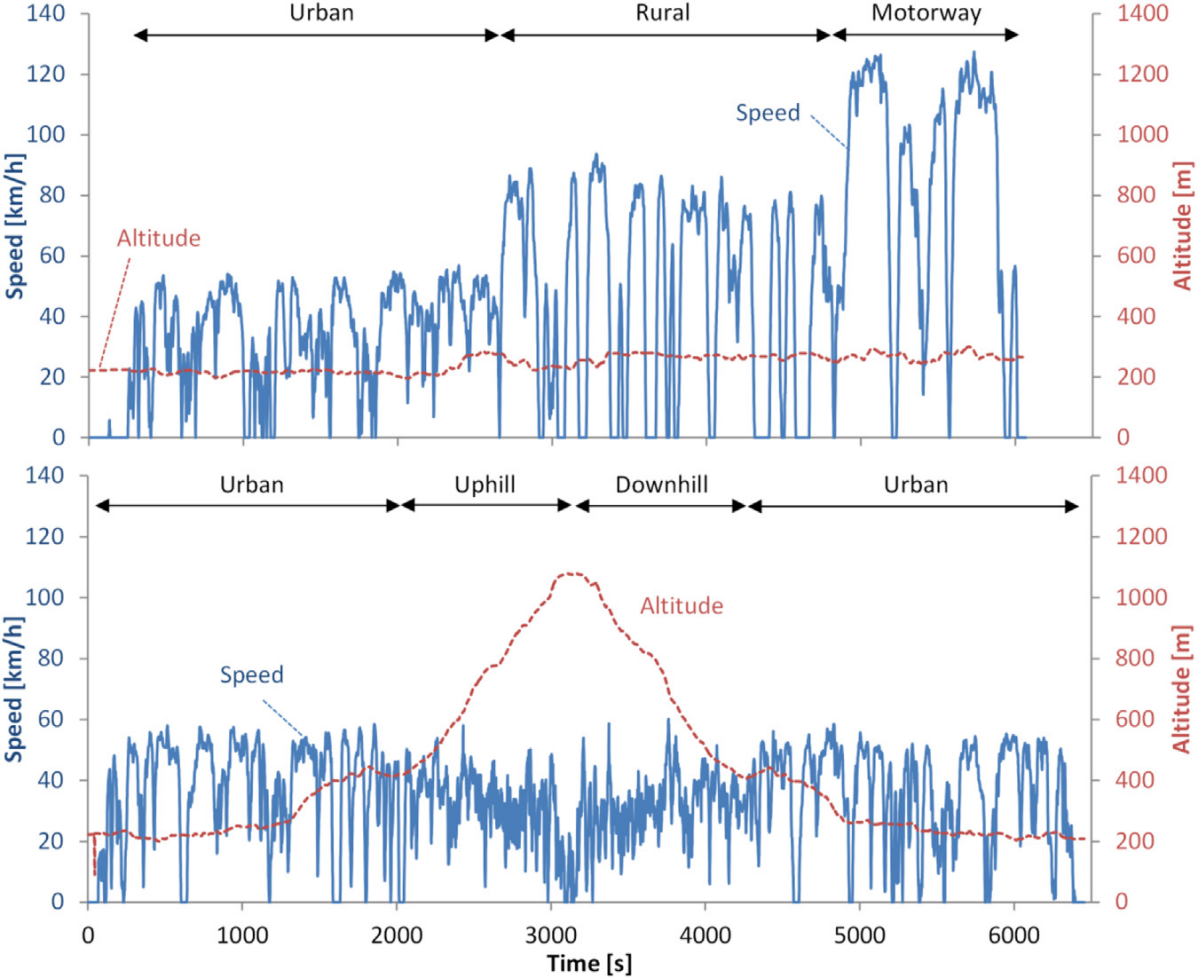
On-road trips. Upper panel: RDE compliant route (Route 1). Lower panel: High altitude route (Route 2). The official route for the prize was the “Urban” and “Rural” parts of Route 1.

The Portable Emissions Measurement System (PEMS) that was used to measure CO_2_, CO and NO_x_ (with two separate chemical cells for NO and NO_2_) during the on-road tests was the Ecostar from Sensors (MI, USA) (Sensors, 2011). The first route, which complied with the trip requirements defined in the RDE regulation (EU, 2017a) (Table 1), was carried out in the morning starting with cold engine (Fig. 3). The second route, which was carried out after a 2 h break, was not RDE compliant, but focused on urban conditions and high altitude (1100 m) (positive altitude gain 1800 m per 100 km) (Table 1). The vehicle battery was left to recharge before each test. For the prize, as required in the rules, only the urban and rural parts of the first route were evaluated; here all results will be presented.

**Table 1:** Overview of on-road routes characteristics.

**Route**	**Part**	**Distance [km]**	**Mean speed [km/h]**	**Max altitude [m]**
1	(Cold) Urban	33.0	33	280
1	Rural	28.0	47	280
1	Motorway	27.0	85	300
2	Urban	20.5	36	450
2	Uphill	9.0	32	1100
2	Downhill	9.0	32	1100
2	Urban	21.5	34	450

The car was parked indoors at a temperature of 16 °C. The ambient temperature during the tests was 6–10 °C and the relative humidity 65–95%. The weight of the car with the instruments, the driver and the co-driver was 1650 kg.

The same market quality diesel fuel (< 7% biodiesel content, 8 ppm sulphur) was used for all tests both in the laboratory and on the road. The same person drove both in the laboratory and on the road.

### 3.4 Quality checks

The agreement between the different analysers was good. Regarding CO_2_, the bag and diluted results agreed within 2%. The tailpipe, PEMS (validated once) and FTIR results were typically within 4% from the bag results. Regarding NO_x_, the bag, diluted, tailpipe and PEMS results were within 10% (or 15 mg/km for levels < 150 mg/km). The FTIR NO_x_ emissions had 4% ( ± 9%) difference from the tailpipe and 11% ( ± 10%) from the bags. These differences are similar to the differences found in a study that compared NO_x_ emissions from various techniques (including those used in this study) (< 17% for tailpipe CLD, < 11% diluted CLD, < 13% for PEMS, < 10% for FTIR) (Olsen et al., 2010). In our study, additionally other uncertainties, for example, due to the exhaust flow rate used for the PEMS and FTIR probably contributed to the observed differences. Nevertheless, the differences are well within the permissible tolerances of the regulated validation check (10% for CO_2_ and 15% for NO_x_).

## 4 Results

The results of the tests in the laboratory and on the road will be presented in the next sections.

### 4.1 Regulated pollutants (chassis dynamometer)

**Fig. 4:**
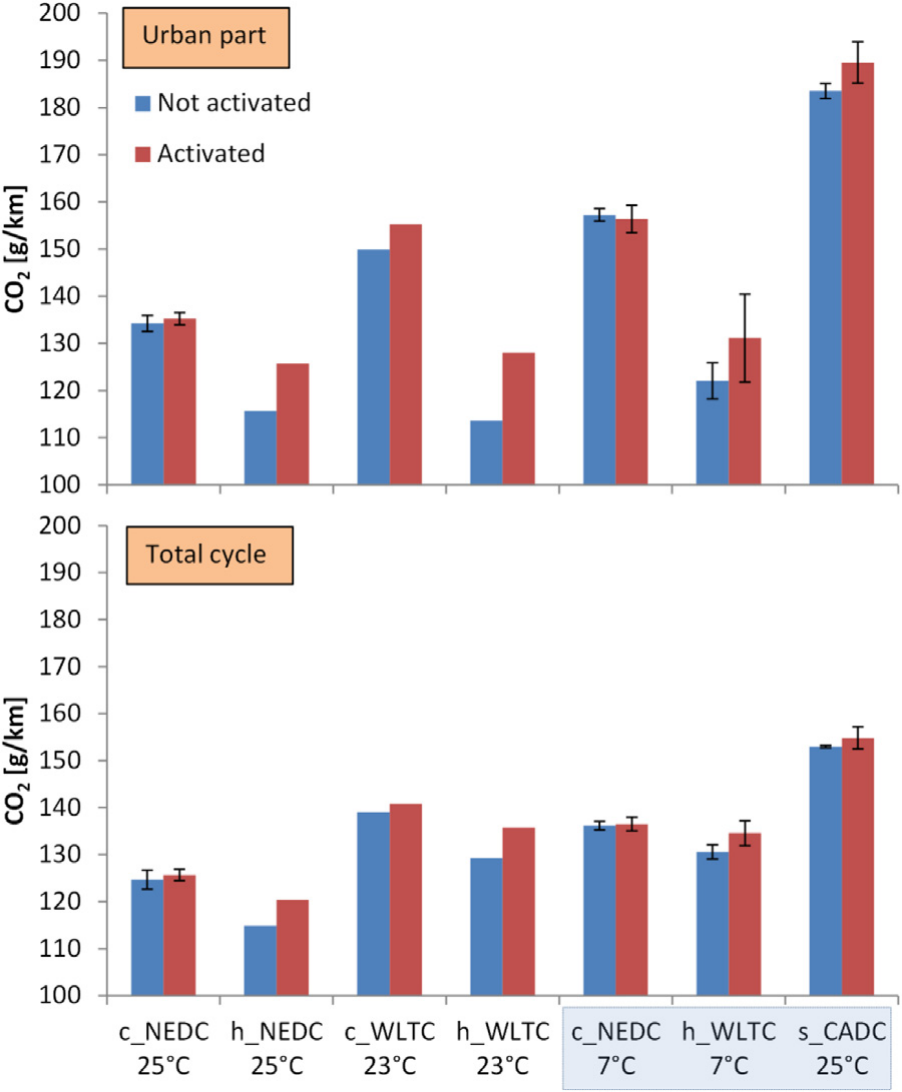
CO_2_ emissions with the retrofit activated or not activated for various test cycles. Upper panel: Urban part emissions. Lower panel: Total cycle emissions. Asterisk * indicates type approval road loads. Error bars show the difference between maximum value and mean value when 2 or more repetitions were available. Cycles for the prize in a box.

Fig. 4 presents the results of CO_2_ emissions for the vehicle with or without activation of the retrofit system for various test cycles (NEDC, WLTC or CADC), with engine cold or hot start, and at two ambient temperatures (7 °C or 23–25 °C). The results for the whole cycle (lower panel) or only the urban part (upper panel) are separately plotted. For the cold start tests, where the vehicle battery was fully charged, the influence of the retrofit on CO_2_ is negligible. For the hot start tests, the retrofit increases the CO_2_ emissions between 4 and 7 g/km, with one exception (hot start WLTC urban part) which had higher (+15 g/km) difference.

**Fig. 5:**
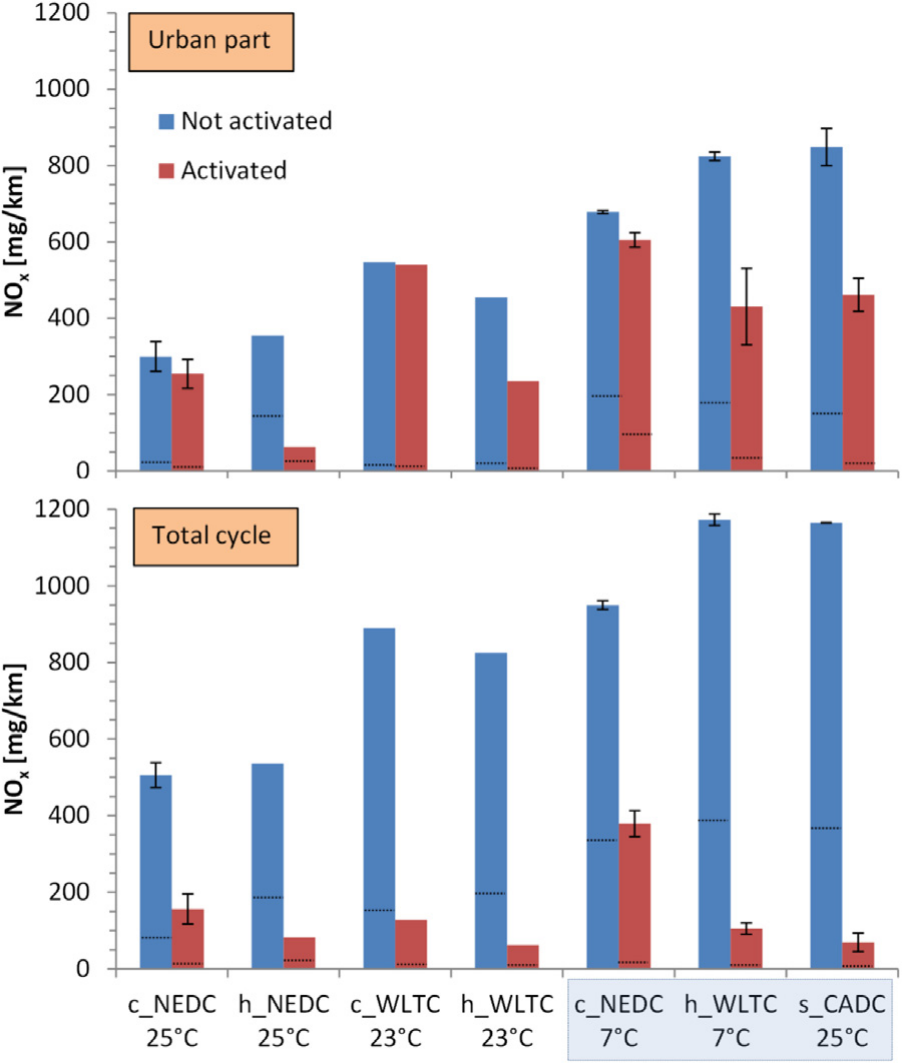
NO_x_ emissions with the retrofit activated or not activated for various test cycles. Threshold of total cycles:180 mg/km. Upper panel: Urban part emissions. Lower panel: Total cycle emissions. Asterisk * indicates type approval road loads. Error bars show the difference between maximum value and mean value when 2 or more repetitions were available. Dashed lines show NO_2_ emissions (low part of the bar). The rest is NO. Cycles for the prize in a box.

Fig. 5 presents NO_x_ emissions for various cycles. The NO_x_ emissions without the retrofit activated are very high, exceeding the 180 mg/km certification limit, except the cold NEDC at 25 °C with the type approval road loads, reaching 1100 mg/km in the hot WLTC or CADC.

For the urban part of the cycles the decrease of the NO_x_ emissions with the activation of the retrofit is negligible for some cold start tests (e.g. cold NEDC) but significant for some hot start tests (e.g. hot WLTC). The reduction of NO_x_ is very high for the complete cycles with emissions > 500 mg/km, both as absolute reduction (350–1100 mg/km) and relative reduction (60–95%). Regarding the prize rules the retrofit solution achieved a significant reduction of the mean emissions of cold NEDC (7 °C), hot WLTC (7 °C), and CADC (25 °C) to just below 180 mg/km (from 1100 g/km).

With active retrofit the NO_2_ decreased to levels < 25 mg/km for the complete cycles with relative reductions over 88%. The mean ratio NO_2_/NO_x_ for all cycles was 27% (16–40%) with the retrofit deactivated and dropped to 10% (2–25%) when the retrofit was activated.

**Fig. 6:**
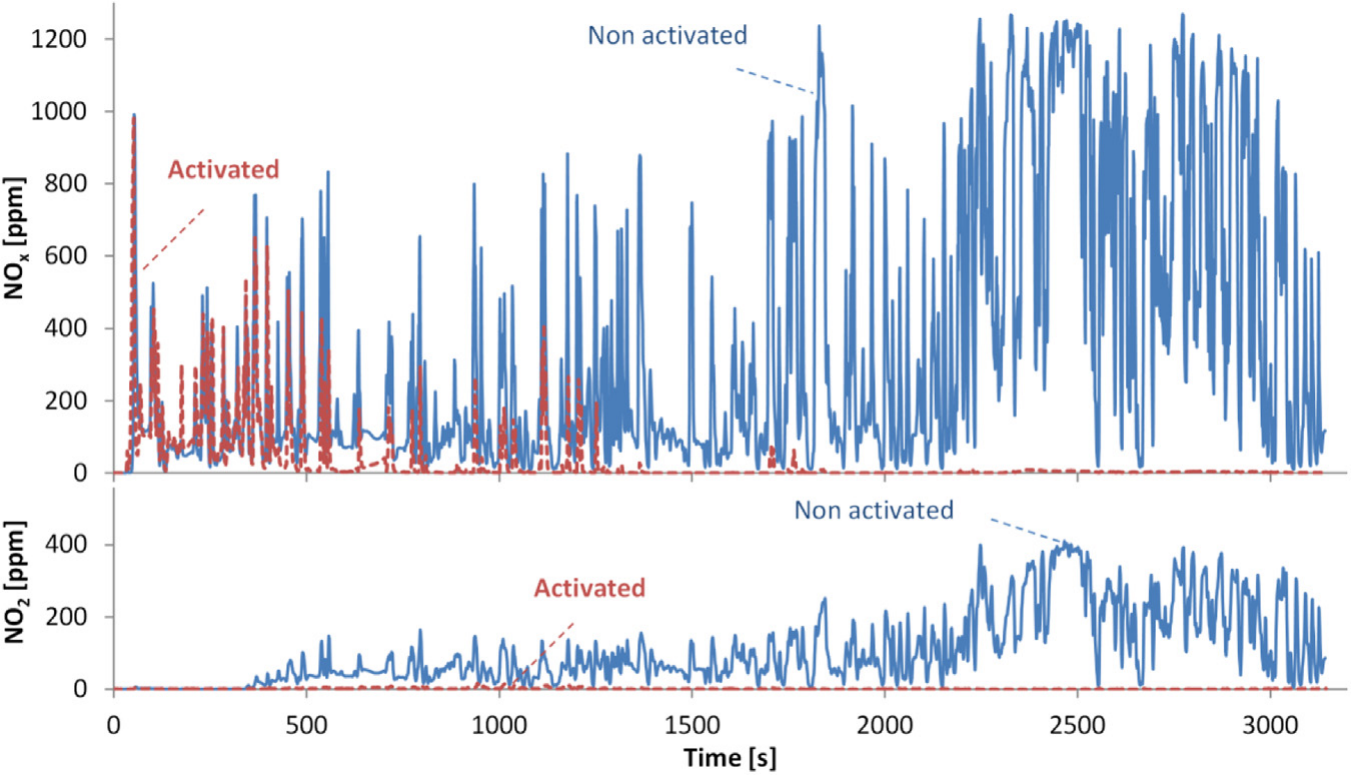
Real time NO_x_ (upper panel) and NO_2_ (lower panel) concentration (at the tailpipe) for CADCs with semi-cold engine start (25 °C).

The variable NO_x_ reduction can be explained with an example. Fig. 6 plots the real time NO_x_ concentrations at the tailpipe of the vehicle of the (semi-cold start) CADC with the retrofit activated or not activated. At the beginning of the cycle the exhaust gas temperature and therefore the SCR temperature in under-floor position, is low and the light-off of the SCR catalyst becomes a limiting factor even though the start-up unit is already ready to dose. As the exhaust gas temperature increases, and the NO_x_ sensor upstream of the SCR catalyst reaches 140 °C, at approximately 400 s, ammonia dosing commences and the NO_x_ emissions start to decrease. After 1250 s the NO_x_ concentration is at almost zero levels. The same figure (lower panel) also shows NO_2_ concentrations. They increase as the exhaust gas temperature increases as the NO to NO_2_ conversion at the DOC increases, probably to assist the passive DPF regeneration with NO_2_ at lower temperatures. With the retrofit activated NO_2_ is almost completely reduced because at these exhaust gas temperatures the reduction efficiency of the SCR is optimal including the effect of the fast SCR reaction involving NO and NO_2_ (Goo et al., 2007).

Regarding PN emissions, they were in general low (< 10^10^ p/km). Only during cold start tests (NEDC at 7 °C) the emissions were high (>10^11^ p/km) exceeding the PN limit (6 × 10^11^ p/km). There was no negative effect of the retrofit on PN: the emissions were even slightly lower, although this probably has to do with the DPF fill state rather than the introduction of the SCR reductant. Most importantly, the retrofit did not increase the PN concentration of sub-23 nm particles, a recent concern for heavy-duty vehicles equipped with SCR systems (Giechaskiel, 2018).

The rest of the pollutants (CO, THC) were also low and unaffected (or slightly improved) by the retrofit. But in any case the emission levels were much lower than the emission limits set in the prize rules.

### 4.2 Unregulated pollutants (chassis dynamometer)

**Fig. 7:**
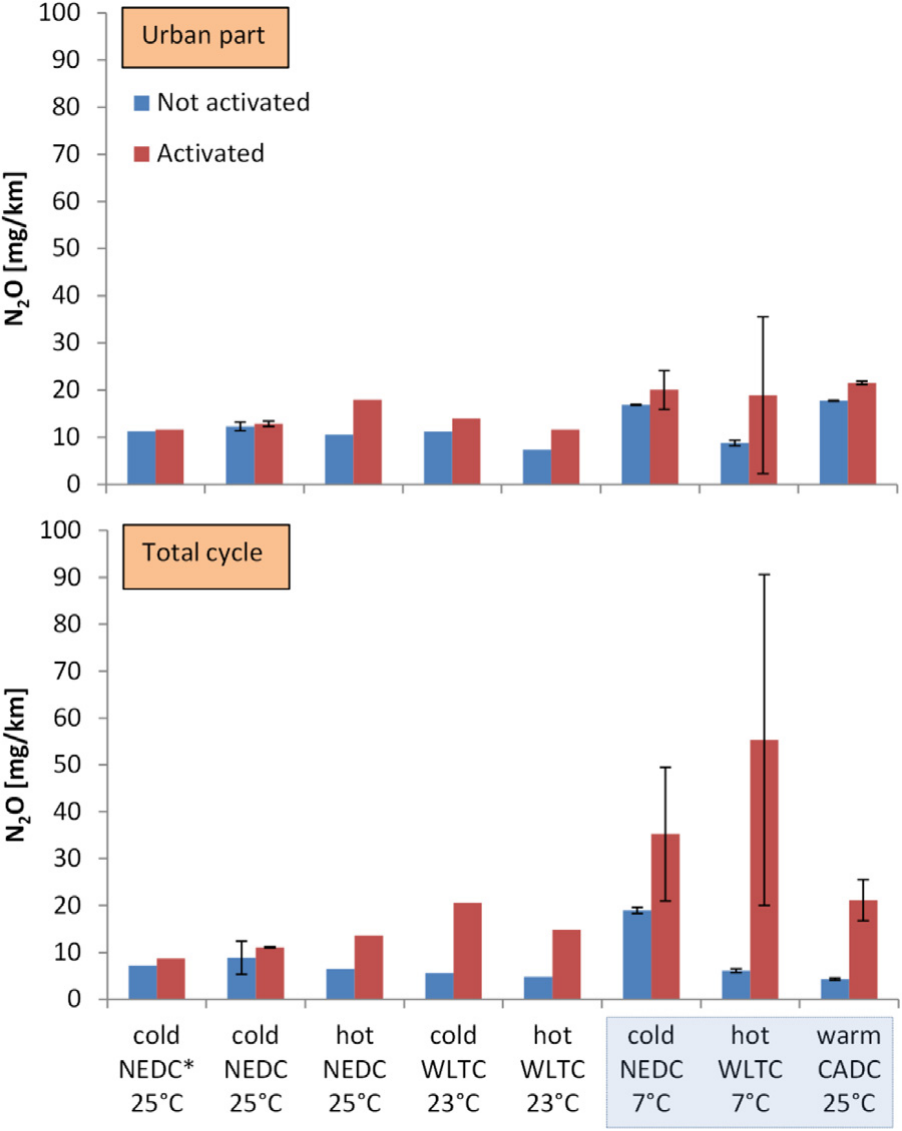
N_2_O emissions with the retrofit activated or not activated for various test cycles. Threshold: 40 mg/km. Left panel: Urban part emissions. Right panel: Total cycle emissions. Asterisk * indicates type approval road loads. Error bars show the difference between maximum value and mean value when 2 or more repetitions were available. Cycles for the prize in a box.

Fig. 7 shows the emissions of N_2_O. For the urban part, N_2_O emissions were visible without retrofit, and turning the retrofit on resulted only in a small increase of the N_2_O emissions (on average +4 mg/km, due to the hot start cycles). For the complete cycles significant increases were seen (on average +16 mg/km), which were even higher for the hot start cycles.

**Fig. 8:**
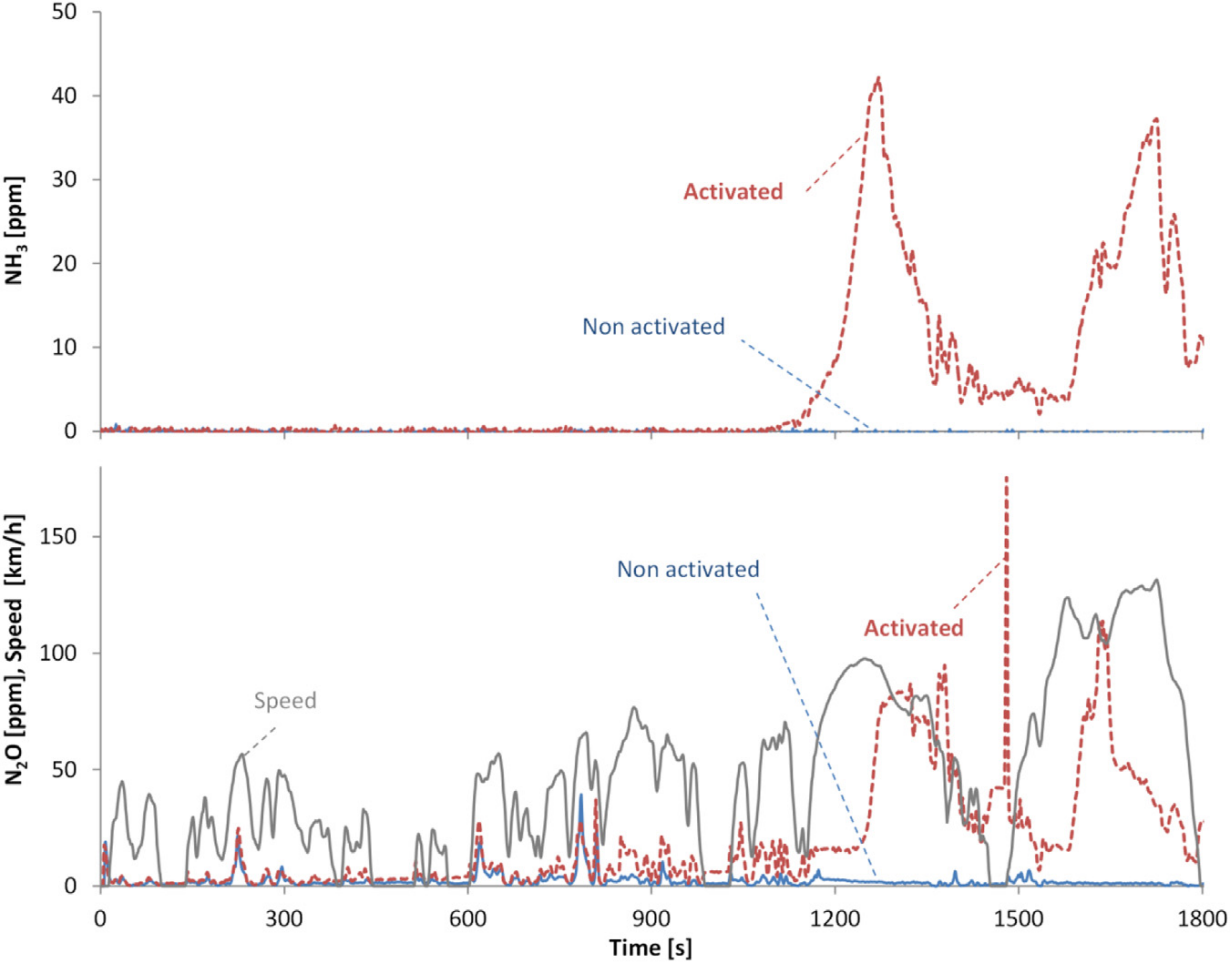
Real time NH_3_ (upper panel) and N_2_O (lower panel) concentration (at the tailpipe) for WLTCs with hot engine start (climatic room temperature 7 °C). Emissions with retrofit activated NH_3_ 11 mg/km, N_2_O 73 mg/km.

Fig. 8 shows an example of the NH_3_ and N_2_O real time concentrations over a hot start WLTC at 7 °C. When the retrofit is active, both NH_3_ and N_2_O increase at around 1200 s, when the “High” part of the WLTC begins and the vehicle accelerates to 100 km/h.

### 4.3 On-road tests

**Fig. 9:**
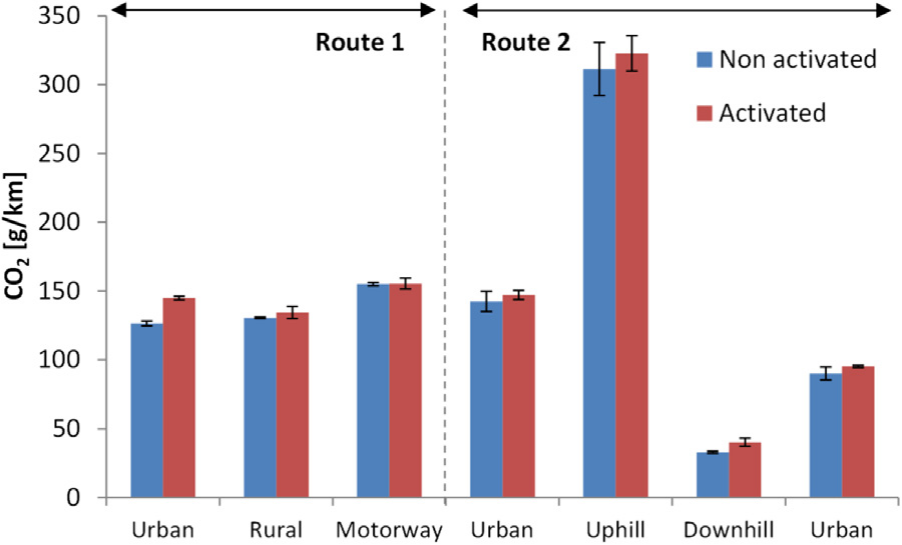
CO_2_ emissions during the real driving emissions testing. The first Urban part is with engine cold start. Error bars show the maximum and minimum value of the 2 repetitions.

Fig. 9 summarises the CO_2_ on-road results over the two performed routes. The emissions range from < 50 g/km (driving downhill) to > 300 g/km (driving uphill). The activation of the retrofit has an influence on the CO_2_ emissions (19 g/km) only in the urban part with cold engine start, probably due to heating of the cartridges for ammonia release.

**Fig. 10:**
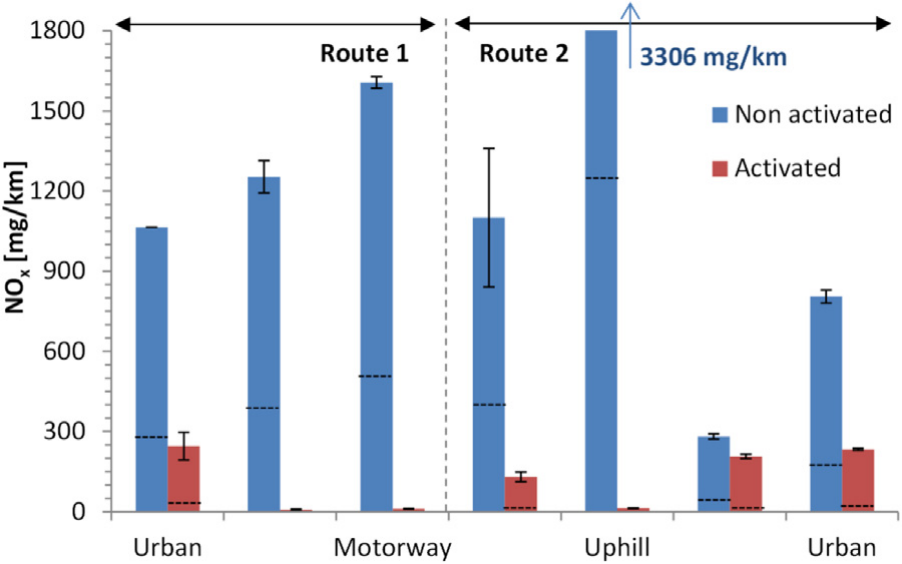
NO_x_ emissions during the real driving emissions testing. The first Urban part is with engine cold start. Error bars show the maximum and minimum value of the 2 repetitions. Dashed lines show the NO_2_ emissions (lower part of bars). Note that the y axis is cut at 1800 mg/km and the “Uphill” emissions exceeded 3300 mg/km.

Fig. 10 shows the results for NO_x_ and NO_2_. The NO_x_ emissions range from 280 mg/km to > 3300 mg/km. The NO_2_/NO_x_ ratio (in ppm) is 18–26%. With the retrofit activated the NO_x_ emissions decrease to < 240 mg/km and the NO_2_/NO_x_ ratio < 15%. The absolute reduction of NO_x_ is 570–3300 mg/km (or the relative > 70%). The only exception is the “Downhill” driving where the reduction is only 75 mg/km (or 26%). Regarding the prize rules, the combined urban and rural emissions with cold start decreased from 991 to 113 mg/km and the NO_2_/NO_x_ (ppm) ratio from 29% to 15%.

The PN results were low (<1.1 × 10^11^ p/km) and there was no effect, within experimental uncertainties, from the retrofit device.

## 5 Discussion

The retrofit system was tested on a Euro 5 vehicle and the results were promising showing a high potential to impact NO_x_ emissions. Below some concerns and open points will be discussed.

### 5.1 NO_x_ emission of the donor vehicle

The emissions of the donor vehicle were just at the Euro 5 limit only with the type approval cycle and road loads (NEDC at 25 °C). However, with the default road loads they were higher than the respective limit (505 mg/km instead of < 180 mg/km). A Bosch diagnostics scan tool found no error messages. The specific vehicle was not recalled by the manufacturer for reprogramming due to high emissions (based on the VIN number), thus it seems that the engine was optimised only for the specific engine map region (type approval conditions). The increase of NO_x_ comes from the EUDC part of the NEDC (the urban parts emissions are similar, see Fig. 5). The aerodynamic resistance with the default values (F_2_) was much higher than the type approval value (0.0502 vs 0.0282 N/(km/h)^2^). The cycle energy demand was estimated to be only 8% higher at the UDC part with the default values, but 34% higher at the EUDC part (equations from Annex 7 of EC, 2017a). The peak power reached 52% of max power with the default values, while 39% with the type approval ones. According to the vehicle manufacturer, the specific family of engines had a calibration error on another vehicle model with similar engine, which were recalled (EMIS, 2016). The only study that we found in the literature on this specific Euro 5 model also found higher emissions (305 mg/km) over the type approval cycle (Kadijk et al., 2016). However, the manufacturer informed the authors of that study (Kadijk et al., 2016) that 14 vehicles of this model had been tested in the frame of an in-service conformity (ISC) program under the control of the type approval authority and complied with the regulation. In any case, the target of the investigation was to see relative changes with the retrofit aiming at a solution to solve the known challenge with high on-road NO_x_ emissions of Euro 5 vehicles.

For other test cycles (WLTC and CADC) at 23 °C the NO_x_ emissions of the donor vehicle increased to > 800 mg/km. Additionally, there was a “thermal window” (17–35 °C) out of which the EGR was significantly reduced and this can explain the even higher emissions over tests performed at 7 °C compared to those at 23 °C (+250 to +450 mg/km). The very high NO_x_ levels, around 1000 mg/km, are in agreement with the findings of the above mentioned study for other than the type approval cycles (Kadijk et al., 2016), and other studies that tested other models from this vehicle manufacturer (Emissions Analytics, 2018). The NO_x_ emissions are at the average-to-high end of the reported values for “real” emissions of Euro 5 diesel vehicles (e.g. see review Hooftman et al., 2018; Ntziachristos et al., 2016).

The ratio NO_2_/NO_x_ ratio was around 27%, within the range reported for other EGR vehicles: 42 ± 19% (O'Driscoll et al., 2018).

### 5.2 NO_x_ reduction of the retrofits

The NO_x_ emission levels of the retrofitted Euro 5 vehicle dropped to < 160 mg/km, with the exception of cold NEDC at 7 °C (380 mg/km). The combustion and EGR strategy of the vehicle, and the different ambient temperatures resulted in high and variable engine out emissions, which challenged the retrofit devices in terms of achieving low absolute levels of NO_x_. The reductions were small for urban phases (1–82% with higher percentages for the hot start cycles). They were high though for the complete cycles (> 65%). The NO_x_ reduction for NEDC with type approval road loads was smaller (31%) due to the initial lower absolute NO_x_ emissions (187 mg/km). The reductions were even higher for NO_2_, the more important NO_x_ pollutant from a health perspective (> 88% for the complete cycles). The reason is that the NO_2_ is formed at the DOC at high exhaust gas temperatures, where the retrofit works efficiently, combined with the better SCR efficiency in presence of NO_2_ (Goo et al., 2007). The lower NO_x_ reduction efficiency at the urban phase and cold start is in line with the dependency of the SCR efficiency with the temperature (e.g. Jung et al., 2017; Lüders et al., 1995). The technical approach of the retrofit having SCR function added downstream of the DPF and avoid any changes to the original engine and aftertreatment configuration puts some natural constraints to the cold-start performance. Even with ammonia dosing from 140 °C, the warm-up of the SCR takes a few minutes.

The amount of stored NH_3_ at the beginning of the test cycle can affect the reduction efficiency of the SCR catalyst (Feng and Lü, 2015). Due to the kinetical limitations of the SCR reactions, stored NH_3_ helps to achieve high NO_x_ conversion, especially in the lower temperature range (< 300 °C) (Yuan et al., 2015). The stored NH_3_ at the beginning of each test was not estimated in this study, but the effect was approached differently: Few hot engine start WLTCs at 7 °C were conducted after the cold engine start NEDC at 7 °C with the retrofit activated or not activated (thus with minimum adsorbed NH_3_ in the second case). The WLTC Low phase (urban) NO_x_ emissions with stored NH_3_ were 430 mg/km, while without stored NH_3_ they were 670 mg/km, and without retrofit 825 mg/km. Although the dosing strategy of the specific vehicle does not take into account the stored NH_3_ with storage model, it uses the NO_x_ concentration measured by the NO_x_ sensors upstream and downstream of the SCR to adjust the dosing. Even though it was optimised for the prize test cycles (WLTC and CADC), these cycles are based on actual driving patterns. Additionally, the good performance of the retrofit was also proved on the road in real PEMS testing.

**Fig. 11:**
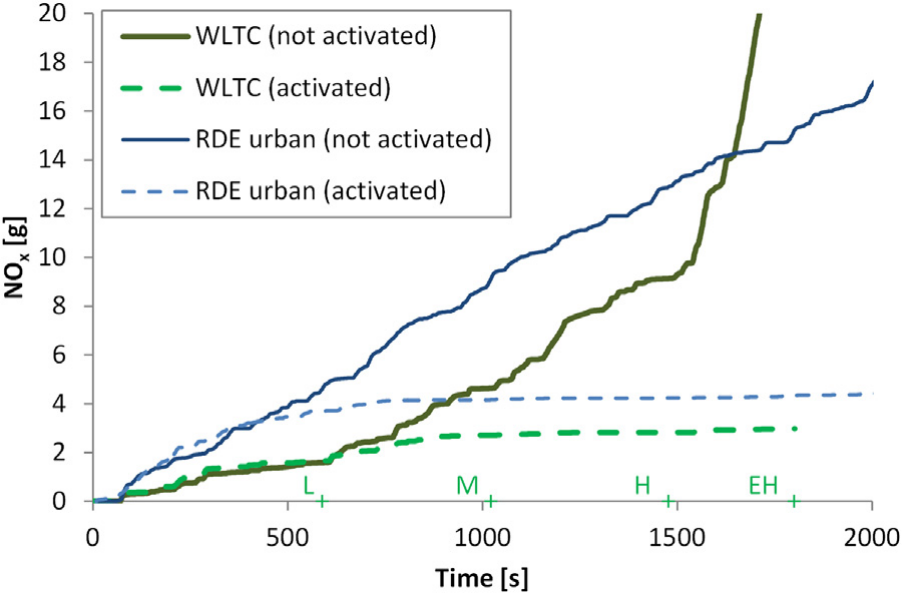
Cumulative NO_x_ emissions for the 1800 seconds of the cold start WLTC tests at 23 °C (thick lines) and the first 2000 seconds of real driving emissions (RDE) tests at approximately 8 °C (urban part) (thin lines). On the x-axis the WLTC phases are also indicated (L=Low, M=Medium, H=High, EH=Extra High).

Fig. 11 presents the cumulative NO_x_ emissions for the vehicle with and without retrofit. The vehicle has an evident (> 10%) decrease of the NO_x_ emissions after approximately 450 s (RDE) to 600 s (WLTC), or approximately 3 km of urban driving. For a specific SCR, the exact timing or distance depends, among others, on the temperature of the aftertreatment device, which is determined by the ambient temperature and the driving pattern at the urban part. For the first seconds, in which the SCR is not working, engine re-calibration (e.g. with software upgrade during the installation of the retrofit) could further reduce the NO_x_ emissions (2–5 g of NO_x_ in the specific vehicles). The reduction of cold start NO_x_ emissions with engine recalibration was recently demonstrated (Triantafyllopoulos et al., 2018). Consequently, the cold-start potential for a retrofit could be improved even further if the vehicle manufacturer would be involved in minor update of the engine control software targeting the first few minutes of a trip.

In order to estimate what would be the mean NO_x_ reduction in cities, one would have to calculate mean trip distances between cold starts. According to a summary report (Weiss et al., 2017), the median distance between two consecutive cold starts is 30 ± 13 km or 27 ± 8 km if only urban trips are considered. Thus, the expected NO_x_ reduction impact of the retrofit solution in cities is similar to the reduction measured at the 23.3 km long cold WLTC at 23 °C (86%) or the urban part (around 33 km) of the RDE (77%).

### 5.3 Unregulated pollutants

The retrofit of the Euro 5 vehicle showed good NO_x_ reduction performance, but under some driving conditions had NH_3_ slip and increase in N_2_O emissions compared to the baseline measurements.

The retrofitted Euro 5 had NH_3_ emissions < 3 mg/km for all cycles except the hot WLTC (10–30 mg/km). Why the emissions are so high only over the WLTC and not the CADC (both in ppm and mg/km) is not clear. One reason could be a not fully optimised calibration. Another explanation is that during the WLTC, which is after the NEDC, stored NH_3_ is additionally released, but this does not happen during the CADC, which is after the WLTC. Complicated models that take into account the stored ammonia, in addition to the exhaust gas temperature and the exhaust flow rate could reduce the NH_3_ slip (Feng and Lü, 2015). In any case, these emission levels of NH_3_ are in agreement with other studies. Although NH_3_ emissions were not an issue in the past for diesel vehicles, with the introduction of NO_x_ reduction systems the levels have increased. For example, modern Euro 6 light-duty diesel vehicles with SCR emit < 7 mg/km NH_3_ (Suarez-Bertoa and Astorga, 2016, Suarez-Bertoa et al., 2017, Vojtíšek-Lom et al., 2018) with some tests reaching 25 mg/km (Vojtíšek-Lom et al., 2018, Suarez-Bertoa and Astorga, 2018). The NH_3_ emissions are not controlled in the light-duty regulations, but there is a limit of 10 ppm (average) for heavy-duty vehicles. For the specific vehicle, the maximum measured value (30 mg/km) corresponded to 20 ppm average NH_3_ concentration at the tailpipe.

The N_2_O emissions were high in the motorway part of all cycles. This increase was likely due to NH_3_ overdosing, and, additionally, at this speed the catalyst reaches temperatures that maximize the production of N_2_O from NO_2_ or NH_3_ (Guan et al., 2014, Jung et al., 2017). It is also possible that when the NH_3_ slip is high the cross-sensitivity of the NO_x_ sensor downstream of the SCR affects the correct dosing (Yuan et al., 2015). The N_2_O emissions increased from 2 to 50 mg/km, which is equivalent to 0.5–13 g/km CO_2_, considering the 265 times higher global warming potential of N_2_O compared to CO_2_ over 100 years (IPCC et al., 2014). Typical N_2_O levels in the literature of Euro 6 diesel vehicles are < 25 mg/km (Vojtíšek-Lom et al., 2018, Suarez-Bertoa and Astorga, 2018), with some tests reaching 45 mg/km (Vojtíšek-Lom et al., 2018). Reviews have reported 10–60 mg/km for diesel vehicles of the 90 s (Lipman and Delucchi, 2002) and < 10 mg/km for Euro 3 and Euro 4 diesel vehicles (Graham et al., 2009). The previous values are in some cases higher than the future limit of 20 mg/km in China (China 6b) from 2020 (ICCT, 2017a) and 10 mg/mi in USA (EPA, 2012).

Reduced motorway emissions would require optimization of the dosing strategy and calibration at high speeds and loads. Future investigations of the SCR and ammonia slip catalyst material and volume could further reduce the non-regulated pollutants.

### 5.4 Durability

The durability requirements of retrofit devices for heavy-duty applications are covered in UNECE Regulation 132 (UNECE, 2014). The retrofit should have a useful life of at least 6 years or 4000 h or 200,000 km. Durability of retrofit devices for passenger cars is not regulated yet.

The durability of the whole retrofit system was not validated. One part of it (ASDS™) is based on an existing system for heavy-duty applications, and there is a lot of experience on the field since 2015 with hundreds of Euro IV/V buses (Johannessen, 2017). The other main part, the SCR, is a conventional Euro 6 catalyst technology which is fitted to several production light-duty vehicles and should therefore fulfil the durability requirements of the regulation (160,000 km) (UNECE, 2015). However, the accelerated ageing procedure for light-duty diesel vehicles has not been thoroughly assessed and validated, especially for NO_x_ reduction technologies (Galassi and Martini, 2014). Lastly, the sensors added to the exhaust (NO_x_ and temperature sensor before the SCR catalyst and NO_x_ sensor after the SCR catalyst) are standard validated automotive parts already used in the used in Euro VI and Euro 6 solutions on the market.

### 5.5 Safety

The solid form of ammonia, AdAmmine™, is confined in closed metal cartridges, which are then placed in the ASDS™ system. At room temperatures, the equilibrium pressure is below 0.6 bar, thus the ASDS™ complies with all transport and storage regulations (UN, 2015). The ASDS™ cartridges can be stored or transported safely at temperatures ranging from -40 to + 80 °C.

An ammonia detector is installed in the trunk compartment; in case of even a small leak with an activation of the ammonia sensor (at 100 ppm), the power to ASDS™ system is cut off. The ASDS™ system is installed in the trunk in the spare-wheel location with a plate fixed on top of it and sealing around. A sealed window in the plate, allows access to the cartridges. Additionally, natural ventilation in the bottom / side of the spare wheel well, would vent any ammonia in case of a leakage. A controlled ammonia leak test by the manufacturer showed that when more than 350 ppm were measured below the plate, no ammonia was detected in the trunk or cabin. Nevertheless, ammonia at low concentrations (10 ppm) has a strong smell before it becomes irritating or a safety hazard. According to the manufacturer, the system passed a drop test (3 m), fire test on the complete system and on the cartridges, crush test, and maximum pressure test by heating (Johannessen, 2017). A study of solid materials as alternative ammonia sources concluded that for the most likely leak scenarios, ammonia concentrations would not pose a significant safety hazard (Fulks et al., 2009).

The cartridges new as well refilled are provided by the manufacturer. When a cartridge is depleted, it is exchanged with a refilled unit. Separately, ammonia is reabsorbed in the cartridge via a refill process by the manufacturer. However, for a large roll-out, the cartridges for the required infrastructure supporting the vehicle owners could be provided in partnership with leading industrial gas companies already having ammonia distribution centres where cartridge refill process could be implemented regionally.

### 5.6 Costs and fuel consumption

According to the prize rules, the (acquisition) cost of the retrofit system plus running costs (consumables and increase of fuel consumption) should be < 2000 Euros for 100,000 km. The applicant estimated them around 1750 Euros for a significant volume of at least 50.000 units per year.

It is difficult to evaluate the market cost when official requirements are not established combined with variation between different vehicle models. A study estimated a mass-market (hardware) cost of 400–600 $ for a vehicle manufacturer to include a SCR NO_x_ reduction system (Posada Sanchez et al., 2012). That study included the catalyst, urea tank, urea pump and injector, urea-exhaust mixer, temperature sensor, urea level sensor and housing. Additional cost for NO_x_ sensor for optimization of urea injection and/or an ammonia slip catalyst were not included but they are expected to increase cost by 10–20%. Another study reported costs for retrofitting between 1400 and 3300 Euros per car (ADAC, 2018).

The cost of consumables (AdAmmine™ cartridges) for 100,000 km was estimated by the applicant to be around 200 Euros, assuming 15 Euros per refilled cartridge and 6.5 refills of the pair installed in the car over the total distance; a cost comparable to the conventional way of vehicle owners purchasing AdBlue today. The intended solution would consist of two intermediate-sized cartridges (2 × 4.5 l AdAmmine™ material) containing approximately 3.4 kg ammonia, which should be equivalent to 17 l of AdBlue. This quantity of AdBlue is, enough for 15,500 km (assuming an equivalent 1.1 litres of AdBlue per 1000 km) (Kadijk et al., 2015) or half for high emitting vehicles such as those in this study. For the donor vehicle and prototype system (with two 1.2 l cartridges) the autonomy would be around 2000 km. At our tests it was less, probably due to not fully calibrated NH_3_ empty-detection algorithms.

The cost of the fuel penalty due to the retrofit was estimated assuming fuel consumption of 5 l/100 km, 1.1. Euros per litre as diesel cost, and 1% fuel penalty. The fuel penalty, which should be < 10% according to the prize rules, was estimated by the applicant at around 1%, mainly due to the use of electrical power for heating the cartridges (peak 300 W, 30–80 W average) for the controlled release of ammonia. This consumption is in line with the estimated power required for light-duty urea SCR applications in the literature (100–200 W) (Van Schaftingen and Op de Beeck, 2006)).

Based on the World Harmonised Light-duty Test Procedure (WLTP) regulation (EU, 2017a), the increase of CO_2_ for a specific test cycle can be estimated based on the equation of the energy used from the vehicle battery:

$$\Delta {Μ}_{CO2}=0.0036\times E\times W_{f}/n/d$$

Where *ΔM*_*CO2*_ is the resulting CO_2_ mass emission difference for the considered period assuming that the necessary energy *E* (Wh) was taken from the battery and the battery had to be re-charged during the tests. *W*_*f*_ is the Willans factor for a diesel vehicle (161 gCO_2_/MJ), *d* is the distance (km), *n* is the alternator efficiency (0.67).

Assuming a mean consumption of 100 W for the respective times and a distances of the three official tests (cold NEDC and hot WLTC at 7 °C and CADC at 25 °C), the CO_2_ difference is estimated 2.6 (NEDC), 1.9 (WLTC) and 1.4 (CADC) g/km. The weighted CO_2_ increase is then calculated 1.7 g/km, while the experimental was 2.3 g/km for the Euro 5 vehicle. The same results were also calculated using the equation for electrical components from the eco-innovations guide (EU, 2017b).

It should be noted though, considering the high NO_x_ reduction achieved by the retrofit, the 1–2% CO_2_ impact of is probably low compared with level of fuel penalty linked to a major engine/EGR recalibration to achieve similar levels of NO_x_ reduction. A study reported approximately 7% fuel penalty when going from a Euro 5 calibration (level 180 mg/km) to a Euro 6 (level 80 mg/km) (Johnson, 2009).

## 6 Conclusions

This paper assessed a retrofit device on a Euro 5 with EGR, DOC and DPF. The NO_x_ emissions of the vehicle were reduced on average from 865 mg/km to 150 mg/km (not considering the type approval conditions). It took approximately 3 km of urban driving (450 s) for the Euro 5 vehicle to reach the appropriate catalyst temperature for a considerable NO_x_ reduction. Regarding unregulated pollutants, the N_2_O emissions of the retrofitted vehicle increased on average + 16 mg/km compared to the donor vehicle emissions. The NH_3_ emissions increased only at the CADC and the hot WLTC, while they were practically unaffected at the other cycles.

The retrofit seems not to pose any significant safety hazard. More work needs to be done to assess the durability of the system. Regarding costs, acquisition and installation was estimated to be around 1500 Euros (for large production volumes) and the running costs are expected to be at least 200 Euros for 100,000 km. The fuel penalty was found to be < 2% (around 2 g/km CO_2_) (without considering the N_2_O equivalent CO_2_).

The results of this study demonstrate that retrofitting passenger cars is feasible and can significantly reduce the NO_x_ emissions of vehicles. If the retrofit solution is combined with some level of cooperation with a manufacturer, better cold start performance of the retrofitted vehicles could be achieved (e.g. software upgrade targeting only the cold-start phase). Overall, it is estimated that the air quality of cities can be significantly improved. Standardising the procedure of assessing the retrofit devices is important if more retrofit devices will appear in the market.
